# Baicalein alleviates TNF-α-induced apoptosis of human nucleus pulposus cells through PI3K/AKT signaling pathway

**DOI:** 10.1186/s13018-023-03759-9

**Published:** 2023-04-11

**Authors:** Yang Liu, Dao-kuo Liu, Zhi-wei Wang, Chong Zhao, Jun Miao

**Affiliations:** 1 Graduate School, Tianjin Medical University, Tianjin, 300070 China; 2 Department of Spinal Surgery, Hebei Province Cangzhou Hospital of Integrated Traditional and Western Medicine, Cangzhou, 061013 China; 3 Department of Spine Surgery, Tianjin Hospital, Tianjin University, No. 406 Jiefang South Road, Hexi District, Tianjin, 300211 China

**Keywords:** Baicalein, Apoptosis, PI3K/Akt

## Abstract

**Background:**

Nucleus pulposus (NP) cell apoptosis contributed to disc degeneration. Baicalein, a natural steroid saponin, has been demonstrated to have anti-inflammatory, antiapoptotic, and antioxidative effects in various diseases*.* However, little is known about the roles of baicalein in intervertebral disc degeneration.

**Methods:**

To evaluate the roles of baicalein in disc degeneration and its specific mechanism, human NP cells were incubated with TNF-α and various concentrations of baicalein. Cell viability, extracellular matrix protein expression, catabolic factors, degree of apoptosis, inflammatory factors, and related signaling pathways were evaluated by western blotting, fluorescence immunostaining, TUNEL staining, and reverse transcription PCR.

**Results:**

Baicalein inhibited TNF-α-activated apoptotic signaling and catabolic activity in NP cells. Baicalein promoted PI3K/Akt signaling and attenuated the level of apoptosis-related markers in TNF-α-stimulated human NP cells.

**Conclusion:**

Our work provides that baicalein attenuates TNF-α-activated apoptosis in human NP cells through promoting the PI3K/Akt pathway, indicating that baicalein is a new potential candidate for clinical therapy to attenuate disc degeneration.

**Supplementary Information:**

The online version contains supplementary material available at 10.1186/s13018-023-03759-9.

## Background

The intervertebral disc is an important component of the spinal column, and its dysfunction was the main reason to lead to lower back pain [1]. Low back pain imposed a serious burden on patients’ lives as well as cause very heavy economic and social burden [2]. According to the statistics, at least 70% of people will suffer from low back pain at some point in their life in western countries [3, 4]. Intervertebral disc degeneration is a leading cause of low back pain.

Research on disc degeneration has mainly focused on the changes in metabolites during the stress process of the intervertebral disc itself. It is believed that the abnormal stress and nutritional dysfunction will eventually triggering nucleus pulposus cells apoptosis [5, 6].

NP cells are present in a gelatinous extracellular matrix (ECM) containing collagen II and proteoglycan, which are essential for resistance to compressive axial force of the spine [7, 8]. In healthy NP tissue, the NP cell maintains the metabolic balance of ECM, including aggrecan and collagen with long half-lives [9]. Studies have demonstrated that matrix metalloproteinases (MMPs) and a disintegrin and metalloproteinase with thrombospondin type I motifs (ADAMTSs) disrupt the balance of ECM metabolism during the pathological process of disc degeneration.

Various pro-inflammatory mediators, containing tumor necrosis factor (TNF)-α and interleukin-1β (IL-1β), have been shown to be significantly upregulated in degenerative disc tissue in humans [10, 11]. TNF-α and IL-1β, two essential proinflammatory factors, have been shown to be closely related to the progression of IDD [12]. TNF-α can trigger inflammation, leading to neural swelling and neuropathic pain, and aggravate cell apoptosis due to its cytotoxic effects in humans with lower lumbar pain. IL-1β, the best studied proinflammatory factor, has been shown to trigger the level of various proinflammatory mediators, including TNF-α, IL-6, and several matrix-degrading enzymes, disrupting the balance of ECM metabolism and impairing its turnover in the intervertebral discs [13]. Several molecular pathways, such as nuclear factor-kappa B (NF-κB), mitogen-activated protein kinase (MAPK) and PI3K/Akt signaling pathway, have been shown to be major moderators of IL-1β-induced inflammation and catabolism [14]. The phosphatidylinositol-3-kinase (PI3K)/Akt (PI3K/AKT) pathway is the most commonly altered signaling pathway in human disease. The PI3K/AKT signaling pathway affects many cellular processes including cell proliferation, apoptosis and invasion in a wide variety of cells. Therefore, inhibiting apoptosis of NP may be key for mitigating disc degeneration.

Baicalein, a typical flavonoid compound, is extracted from Radix Scutellariae [15]. Pharmacological works have suggested that baicalein has anti-inflammatory, antiapoptotic, and antioxidative effects in various diseases. For instance, Baicalein was shown to attenuate dimethylnitrosamine-induced acute liver injury by inhibiting apoptosis via reductions in the expression of proapoptotic proteins. Baicalein was also reported to attenuate cerebral ischemia/reperfusion injury via inhibiting toll-like receptor 4 (TLR4) signaling [16].

However, little is known about the potential effects of baicalein on NP cells apoptosis.

Therefore, this study aimed to explore the potential effects of baicalein in TNF-α-treated NP cells and its specific mechanism of action. We hypothesized that baicalein attenuates TNF-α-activated apoptosis in human NP cells through promoting the PI3K/Akt pathway.

## Materials and methods

### Chemicals and materials

**Fig. 1:**
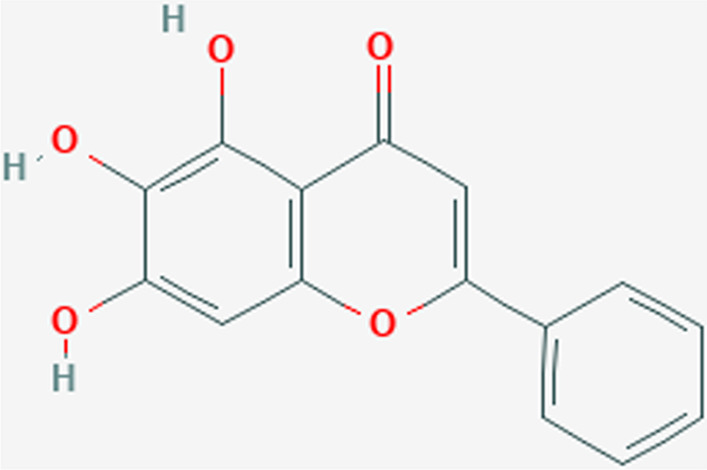
Two-dimensional structure of Baicalein

Baicalein (Fig. 1) with a purity > 99% was purchased from Sigma Chemical Co. (Sigma-Aldrich, Darmstadt, Germany). Baicalein was dissolved in dimethyl sulfoxide (DMSO), with final DMSO concentrations of less than 1%. TNF-α was acquired from R&D Systems (St. Paul, MN, USA). All of the antibodies were acquired from Santa Cruz (Santa Cruz, CA, USA) and Cell Signaling Technology (Beverly, MA, USA). Other reagents not mentioned here were acquired from Solarbio Co. (Beijing, China). This study complies with the World Medical Association Declaration of Helsinki.

### Primary human NP cell culture and treatment

This study was approved by the Ethics Committee of the Tianjin Hospital (CZX2022-KY-011). Informed consent for disc tissue collection was acquired from patients or their relatives. Human nucleus pulposus tissues were derived from 20 patients (6 male patients and 14 female patients; mean age, 45 ± 8.7) with degenerated discs of Pfirrmann level I or II. The protocol of human NP cells culture was performed as described previously [17]. In brief, NP tissues were cut into pieces and enzymatically digested in 0.2% type II collagenase and 0.25% trypsin for 3 h. After filtration through a 70-μm filter mesh to remove cell clumps. NP cells were maintained in DMEM culture medium with 20% fetal bovine serum (FBS). NP cells were stained with toluidine blue, Safranin O and immunohistochemical staining of type II collagen. Identification of NP cells can be seen in Additional file 1. Nucleus pulposus cells were positive for toluidine blue staining (Additional file 1-A), Safranin O (Additional file 1-B) and immunohistochemistry staining of type II collagen (Additional file 1-C).

NP cells at passage 3 were prepared for reverse transcription PCR and western blotting. The cells were planted into 6-well plates. After 2 days, the samples were treated with Baicalein (0, 5, 10, 20, 30, 40, 50 and 60 μM) supplemented with TNF-α (0, 10, 50 and 100 ng/mL) for 1 day, and NP cells were then used for the experiments. The timing of TNF-α intervention for NP cell refers to previously published literature [18].

### Viability assay

The NP cells were seeded and cultured in 96-well plates (6 × 10^3^cells/well) for 1 day and incubated with various concentrations of Baicalein with or without TNF-α (50 ng/mL). After incubation for 1 day, CCK-8 solution (10 μL) was added to each well for 2 h. CCK-8 solution was sterilized by filtration through 0.22-μm-pore-size filters (Costar). The absorbance of sample was tested at 450 nm by a spectrophotometer.

### TUNEL assay

NP cells were seeded and cultured on coverslips, treated with 4% paraformaldehyde for 15 min, and then treated in 0.5% Triton X-100 for 10 min. Following incubation in blocking buffer containing 5% BSA for 30 min, we incubated, fixed, and permeabilized the cells for 1 h with the TUNEL reaction mixture in a humidified atmosphere in the dark. The sample was sealed with VECTASHIELD (Vector Laboratories, Burlingame, CA, USA) including DAPI. The immunostained sample was imaged by laser scanning confocal microscopy. Quantitation of TUNEL labeling is expressed as the number of TUNEL-positive nuclei per area of section, and numbers are an average of at least three independent sections of cells.

### RNA sequencing

There were four biological replicates for TNF-α and TNF-α + Baicalein. RNA was isolated, and concentration was measured according to previously described. RNA sequencing was then performed by Novogene Bioinformatics Technology Co., Ltd (Beijing, China). Differential expression analysis was done using R “limma” package. Meanwhile, volcano plot and heatmap were generated using R software. The function cluster analysis of differentially expressed genes was carried out using Metascape website (http://metascape.org/gp/index.html#/main/step1).

### Predicting targets of baicalein

In STITCH database (http://stitch.embl.de/), the drug’s name (Baicalein) was used for target searching. We set the minimum required interaction score was 0.4000 and *P* value less 0.05 was identified statistically significant. The target proteins were mapped to the Database for Annotation, Visualization and Integrated Discovery (DAVID, https://david.ncifcrf.gov) to enrich gene ontology (GO) and Kyoto Encyclopedia of Genes and Genomes (KEGG) pathway. The filtered *P* value matrix was then transformed by the function *x* =  − log10 (*P* value).

### Western blotting

All protein samples were isolated using cell lysis buffer. The concentration of total protein was tested by the BCA kit. The protein specimens were added onto SDS gel electrophoresis and transferred onto PVDF membrane. After blocking nonspecific binding sites, the membrane was immunoblotted with primary antibodies. On the following day, the sample was treated with the secondary antibody for 60 min at 37 °C. Band was detected using the ChemiDoc XRS + Imaging System.

### Reverse transcription PCR assay

Total RNA of NP cells was extracted using TRIzol method. Reverse transcription was conducted reverse transcriptase amplification kit (Fermentas, New York, USA). Specific reverse transcription primers and quantitative PCR primers were obtained from RiboBio Co. Ltd. (Guangzhou, China). qRT-PCR was performed by the SYBR Premix DimerEraser on a 7900HT system. GAPDH were regarded as the internal references for mRNA. The qRT-PCR results were analyzed by the 2^−ΔΔCt^ method. Primer sequence is shown in Table 1.

**Table 1:** Primer sequence of the target genes

Target gene	Forward (5′–3′)	Reverse (5′–3′)	Product lengths (bp)
Bax	CCCGAGAGGTCTTTTTCCGAG	CCAGCCCATGATGGTTCTGAT	155
Bcl-2	GGTGGGGTCATGTGTGTGG	CGGTTCAGGTACTCAGTCATCC	89
Cleaved cas-3	AAATTGTGGAATTGATGCGTGA	TACAACGATCCCCTCTGAAAAA	164
Cleaved cas-9	GGTCACGGCTTTGATGGAGAT	CCACCTCAAAGCCATGGTCTT	132
MMP-2	TGGCGATGGATACCCCTTT	TTCTCCCAAGGTCCATAGCTCAT	85
MMP-9	TGCCCGGACCAAGGATACAG	TCAGGGCGAGGACCATAGAG	92
GAPDH	AGAAGGCTGGGGCTCATTTG	AGGGGCCATCCACAGTCTTC	258

### Statistical analysis

Results were performed as the means ± standard deviation (SD). Differences among groups were tested by one-way analysis of variance and Tukey’s post hoc test. *P* < 0.05 was regarded as statistical significance.

## Results

### Effects of baicalein on TNF-α-activated cell viability in human NP cells

**Fig. 2:**
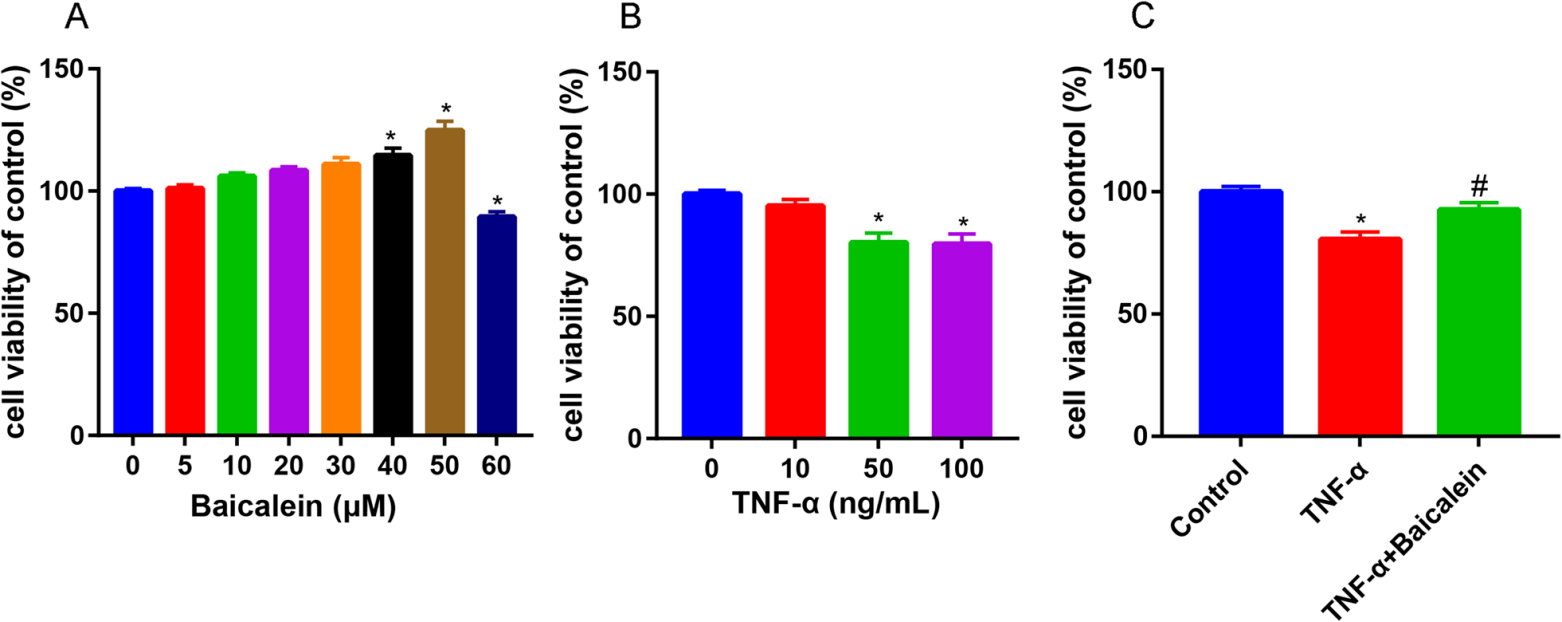
CCK-8 analysis showed that the NP cells viability after treatment with different concentration (0, 5, 10, 20, 30, 40, 50 and 60 μM) of baicalein (**A**, *P < 0.05 compared with 0 μM baicalein) and different concentration (0, 10, 50 and 100 ng/mL) of TNF-α (**B**, *P < 0.05 compared with 0 ng/mL TNF-α). **C** NP cells viability were assessed in the absence and presence of baicalein for 6 h before TNF-α treatment (50 ng/mL), then exposed to TNF-α for 12 h, *P < 0.05 compared with control group, #*P* < 0.05 compared with TNF-α group

We first examined the safe dose range of Baicalein that did not adversely affect the NP cells viability using the CCK-8 method. Baicalein exhibited no effects on cell viability at doses of 5–30 μM (Fig. 2A). A high concentration (> 30 μM) of Baicalein increased the cell viability while a destructive outcome was observed under a concentration of 60 μM. We therefore chose 50 μM Baicalein for further experiments.

NP cells were then incubated with different concentration of TNF-α. However, cell viability decreased with increasing TNF-α concentration (Fig. 2B). The difference of cell viability between the TNF-α of 50 ng/mL and 100 ng/mL was not significant (*P* = 0.307). We chose 50 ng/mL as the TNF-α concentration for further study. Pretreatment with Baicalein partially reversed the inhibitory effect of TNF-α on NP cells viability (Fig. 2C).

### Effects of baicalein on TNF-α-activated apoptotic signaling in human NP cells

**Fig. 3:**
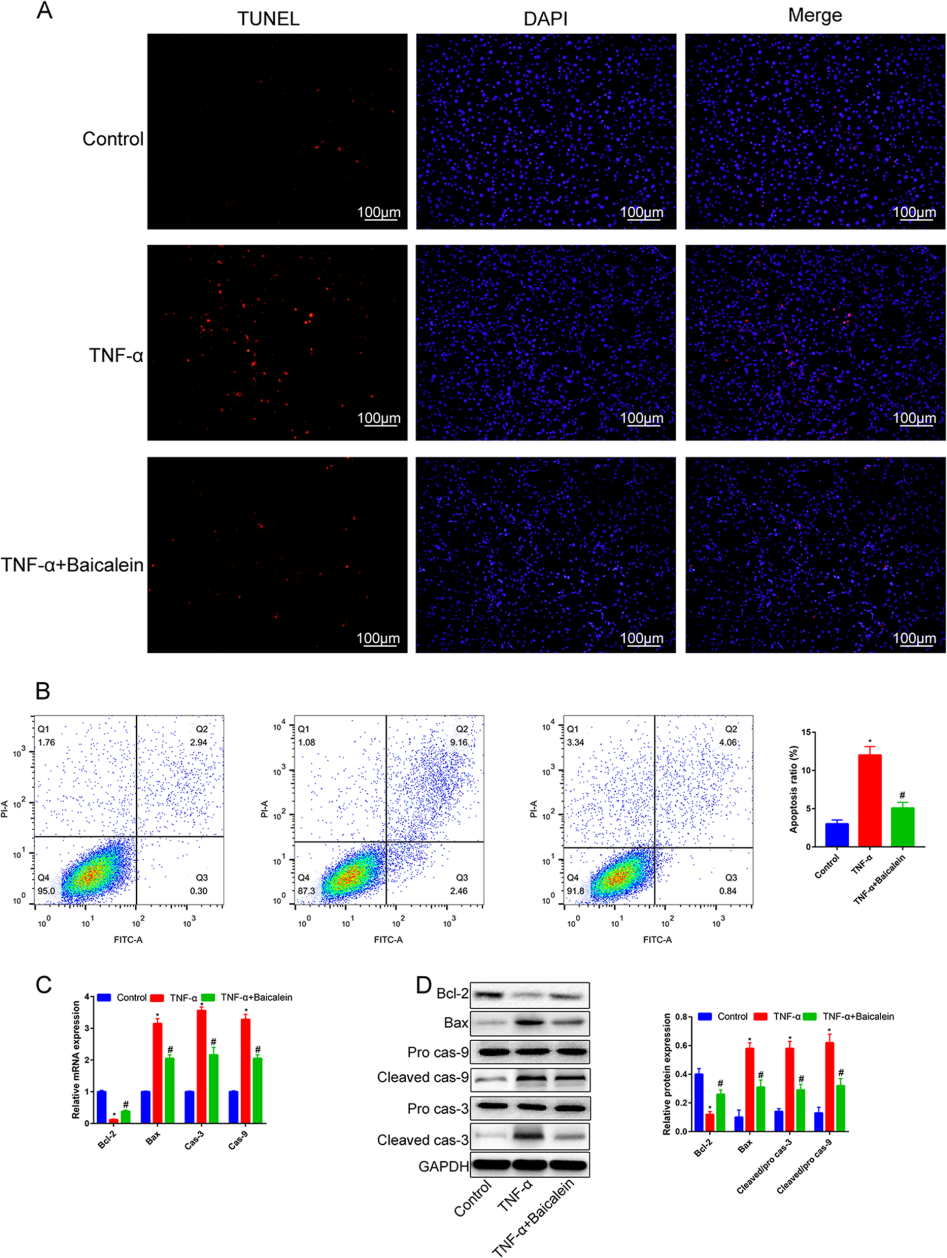
Baicalein inhibits TNF-α-induced NP cells apoptosis. **A** NP cells with or without 50 μM baicalein treatment for 6 h following 50 ng/mL TNF-α for 12 h, as determined by the TUNEL assay. **B** NP cells with or without 50 μM baicalein treatment for 6 h following 50 ng/mL TNF-α for 12 h, as determined by analyzed by flow cytometry after staining with Annexin V-FITC and PI. **C**, **D** mRNA and protein expression of apoptosis-associated genes in NP cells treated in the absence and presence of baicalein for 6 h before TNF-α treatment (50 ng/mL), then exposed to TNF-α for 12 h. *P < 0.05 compared with control group, #*P* < 0.05 compared with TNF-α group

To explore the effect of Baicalein on NP cells apoptosis, we performed a TUNEL assay and found that the percentage of the TUNEL-positive NP cells in the TNF-α is higher than that in the control group. Pre-treatment with Baicalein reduced the TUNEL-positive cell counts (Fig. 3A, *P* < 0.05).

To confirm the above results, Annexin V-FITC/PI assay was then conducted. The rate of apoptosis increased from 3.0 ± 1.0% in the control group to 12.0 ± 1.1% in the TNF-α stimulation group (Fig. 3B, *P* < 0.05). However, after pretreatment with Baicalein, the apoptotic rate decreased to 5.1 ± 0.8%. To further understand the molecular mechanisms underlying the Baicalein mediated apoptosis in NP cells, we investigated apoptosis-related mRNA (Fig. 3C) and protein (Fig. 3D) expression in NP cells. Results indicated that TNF-α significantly increased the expression of the proapoptotic protein Bax, Cleaved caspase-3 and Cleaved caspase-9 and decreased the expression of the anti-apoptotic protein Bcl-2 compared with control group. In Baicalein pre-treatment group, trends in these measures were remarkably reversed. These results suggest that Baicalein may reverse the TNF-α-induced apoptosis through intrinsic apoptotic pathway in NP cells.

### RNA sequencing results

**Fig. 4:**
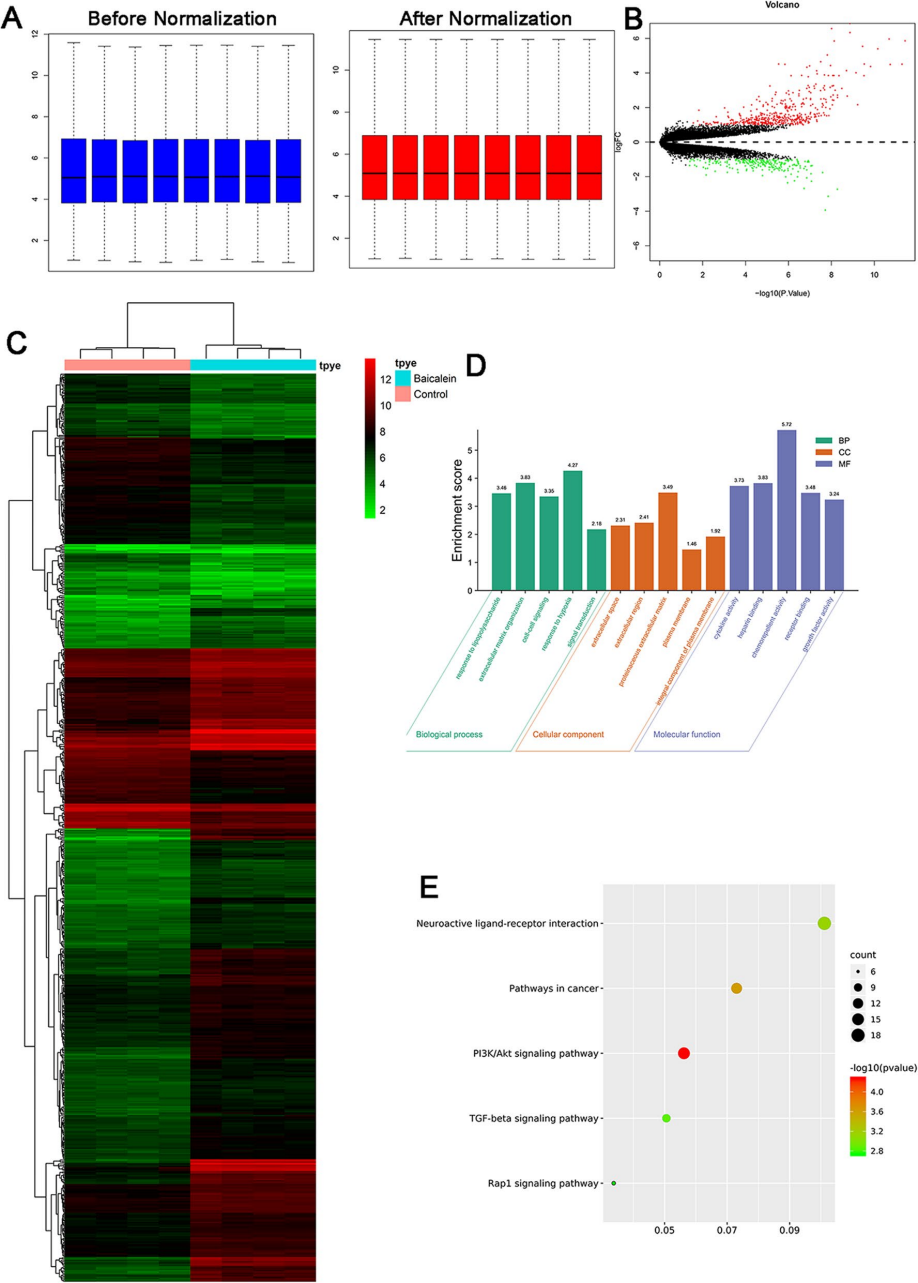
Differentially expressed genes between TNF-α and Baicalein groups. **A** Comparison of expression value between before normalization and after normalization. **B** The volcano plot screen differentially expressed genes between TNF-α and Baicalein groups. **C** The heatmap of differentially expressed genes between TNF-α and Baicalein groups. **D** Gene ontology of differentially expressed genes in TNF-α and Baicalein groups. **E** KEGG pathway of differentially expressed genes in TNF-α and Baicalein groups

Median values in the two groups were almost identical after normalized (Fig. 4A). A total of 697 differentially expressed genes were identified (upregulated = 471, downregulated = 226). The volcano plot and heatmap of differentially expressed genes are presented in Fig. 4B, C, respectively. Furthermore, gene ontology indicated these differentially expressed genes mainly enriched in response to lipopolysaccharide, extracellular matrix organization, cell–cell signaling, response to hypoxia, signal transduction, extracellular space, extracellular region, proteinaceous extracellular matrix, plasma membrane, integral component of plasma membrane, cytokine activity, heparin binding, chemorepellent, chemorepellent activity, receptor binding and growth factor activity (Fig. 4D).

Pathway analysis revealed that differentially expressed genes mainly enriched in PI3K/Akt signaling pathway, Pathways in cancer, Neuroactive ligand-receptor interaction, TGF-beta signaling pathway and Rap1 signaling pathway (Fig. 4E).

### Baicalein suppressed TNF-α-activated apoptotic activity in human NP cells

**Fig. 5:**
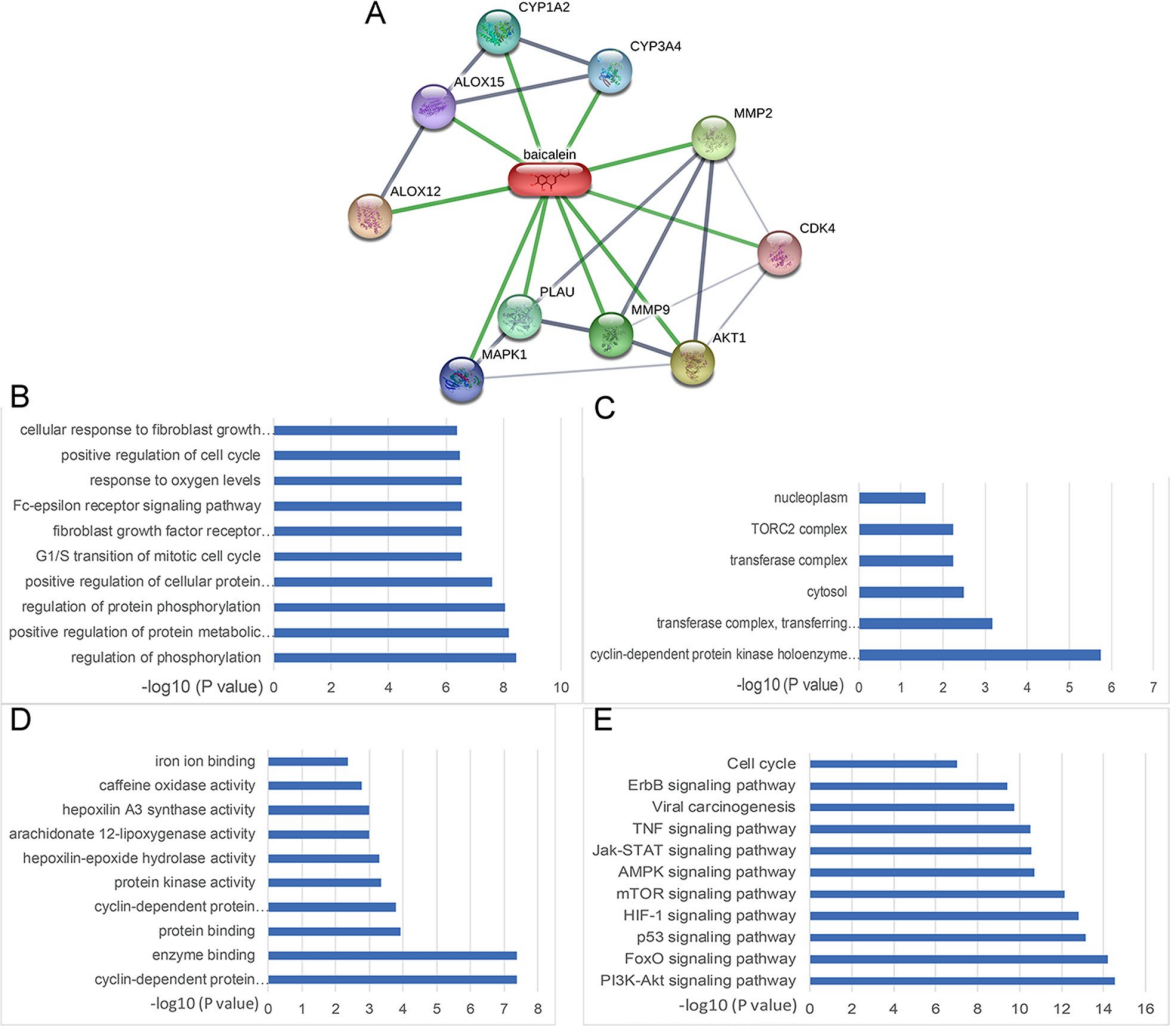
Targeting genes and biological function of baicalein were determined by the STITCH database (http://stitch.embl.de/). **A** A network view of the relationships between baicalein and the target genes; GO enrichment analysis of baicalein target genes **B**–**D** in BP, CC, and MF, respectively. **E** The KEGG pathway enrichment analysis on target genes of baicalein through STITCH database (http://stitch.embl.de/)

Network analysis using the STITCH database revealed tight network among the Baicalein significantly altered proteins (Fig. 5A). Includes the following proteins: CYP1A2, CYP3A4, ALOX15, ALOX12, MMP-2, CDK4, MMP-9, AKT1, PLAU and MAPK1. In total, these altered proteins mainly involved in 10 BP (Fig. 5B), 6 CC (Fig. 5C) and 10 MF (Fig. 5D) terms. The most significant terms of BP, CC and MF enriched by altered proteins were, respectively, regulation of phosphorylation, cyclin-dependent protein kinase holoenzyme, and cyclin-dependent protein. KEGG pathway enrichment analyses were also performed and are presented in Fig. 5E. We found that the PI3K/Akt signaling pathway was the most significantly enriched pathway based on KEGG analysis. Next, we determined if Baicalein play an anti-apoptosis role in NP cells through regulates the PI3K/Akt pathway. We then measured the gelatinases expression (including MMP-2 and MMP-9) in NP cells. PCR results shown that TNF-α administration could significantly increase the mRNA expression of MMP-2 and MMP-9, Baicalein pretreatment significantly reduced the MMP-2 and MMP-9 expression (Additional file 2-A). Western blot results were in accordance with the results of RT-PCR (Additional file 2-B).

### Baicalein attenuates apoptosis in TNF-α-stimulated human NP cells through PI3K/Akt signaling pathway

**Fig. 6:**
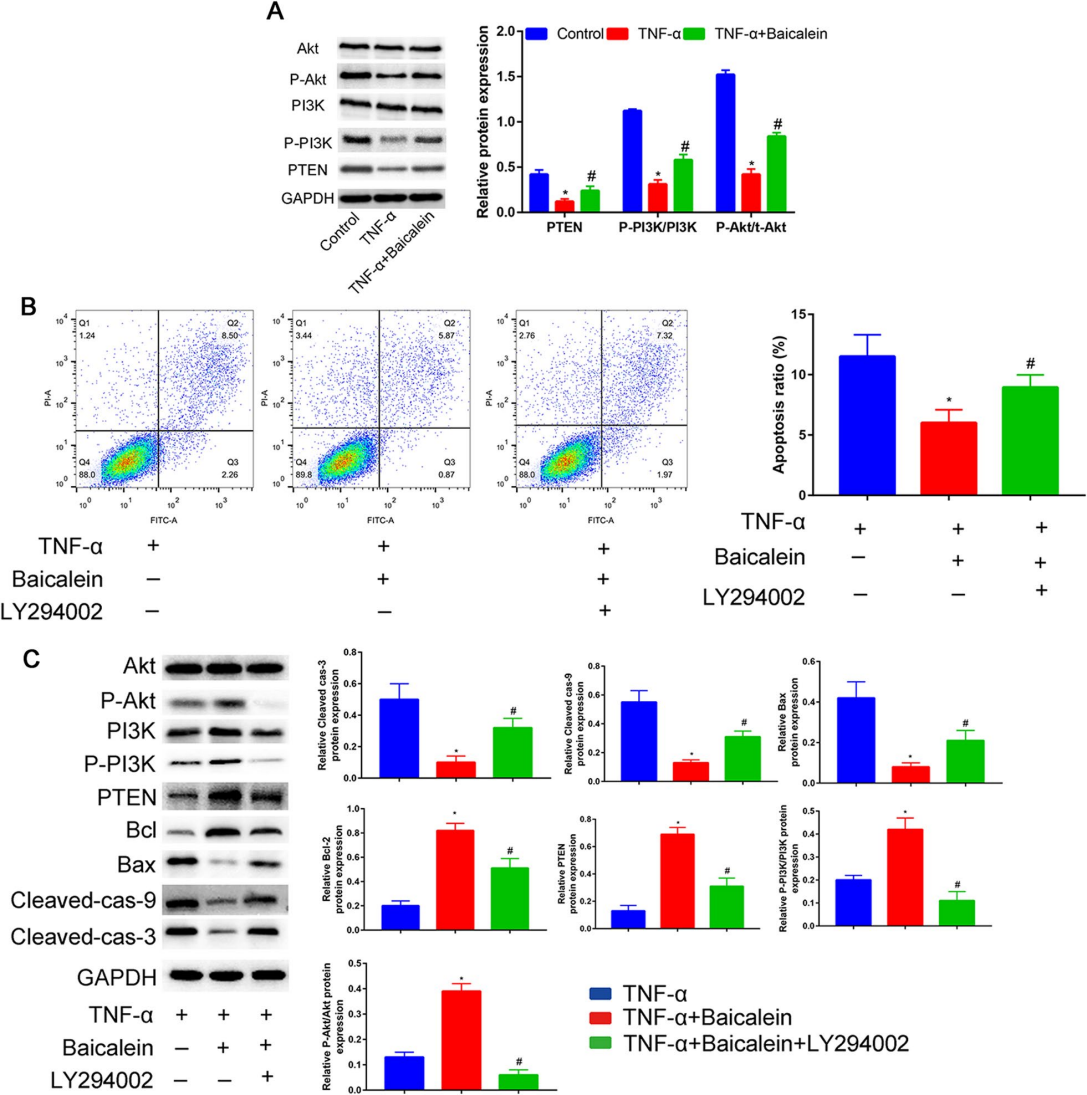
Baicalein modulates apoptosis via the PI3K/Akt signaling pathway. **A** Western blot analysis was used to determine the PI3K/AKT pathway proteins; **B** NP cells were treated by Baicalein with TNF-α at a concentrations 50 ng/mL, and with or without LY294002, and the apoptosis of NP cells was detected by the Annexin-V-FITC/PI double staining method. The proportions of dead cells (Q1: Annexin V-FITC−/PI+), late apoptotic or necrotic cells (Q2: Annexin V-FITC+/PI+), early apoptotic cells (Q3: Annexin V-FITC+/PI−) and live cells (Q4: Annexin V-FITC−/PI−) are displayed. **C** PI3K/Akt signaling pathway related protein expression levels and apoptosis-related protein expression were analyzed by western blot assay. *P < 0.05 compared with TNF-α group, #*P* < 0.05 compared with TNF-α + Baicalein group

To test whether Baicalein attenuates apoptosis in TNF-α-stimulated human NP cells through PI3K/Akt signaling pathway. We first compared the levels of PI3K and Akt and phosphorylated PI3K (p-PI3K) and Akt (p-Akt) in TNF-α stimulated alone or with Baicalein (Fig. 6A). PI3K and Akt phosphorylation was increased after TNF-α treatment and Baicalein pretreatment significantly reduced this phosphorylation. Finally, we examined the expression levels of PTEN, which is the upstream regulatory of PI3K/Akt signaling pathway. We revealed that baicalein pretreatment could reverse the inhibitory effects of TNF-α on the PTEN expression.

Next, we performed an experiment using LY294002, an inhibitor of the PI3K/AKT pathway. These anti-apoptotic effects of baicalein were partially reversed by pretreatment with LY294002 (Fig. 6B).

We found that inhibition of PI3K and Akt phosphorylation with a PI3K specific inhibitor (LY294002) substantially abrogated PI3K and Akt phosphorylation induced by Baicalein (Fig. 6C).

Western blotting indicated that pretreatment with LY294002 in baicalein group significantly increased the expression of the proapoptotic protein Bax, Cleaved caspase-3 and Cleaved caspase-9 while decreased the expression of the anti-apoptotic protein Bcl-2. These data suggest that Baicalein prevents TNF-α-induced apoptosis through PI3K/Akt signaling pathway.

## Discussion

Studies have suggested that inflammation and apoptosis are two essential characteristics in NP tissue during the pathological process of IDD [19]. In healthy NP tissue, the NP cells maintain the metabolic balance of ECM, including aggrecan and collagen, with long half-lives [9]. A recent study showed that apoptosis of NP cells is closely related to ECM degradation [20]. Furthermore, inflammatory cytokines (IL-1β and TNF-α) attract death-related signaling complexes through interaction with their specific ligands, and then initiate apoptotic signaling, contributing to DNA fragmentation [21].

It was reported that Baicalein protected PC12 cells against Aβ_25-35_-induced cytotoxicity via inhibition of apoptosis [22]. Baicalein was also shown to alleviate liver oxidative stress and apoptosis induced by high-level glucose through activation of the PERK/Nrf2 signaling pathway [23].

The results of the present work suggested that TNF-α promotes NP cell apoptosis, which is partly inhibited by baicalein. These results suggest that Baicalein exerts an antiapoptotic effect on NP cells in an inflammatory environment. Another strength of this study was that we used STITCH database to predict candidate target proteins of Baicalein.

To better understand the potential biological functions of these candidate proteins, we performed the gene ontology (GO) and KEGG analysis. These proteins mainly participant into regulation of protein phosphorylation and PI3K/Akt signaling pathway was the most enriched pathway.

The PI3K/AKT pathway is involved in protecting cells from apoptosis has been reported in many previous studies [24–26]. First, we found that baicalein enhanced PI3K and Akt phosphorylation level in TNF-α stimulated NP cells. Wang et al. [27] also revealed that baicalein could also stimulate PI3K and Akt phosphorylation in undifferentiated thyroid cancer cells. Similar to our research, baicalein improves glucose metabolism in insulin resistant HepG2 cells through PI3K/Akt signaling pathway.

## Conclusion

PI3K/Akt signaling was suggested to be related to the mechanisms of action of baicalein against TNF-α-activated apoptosis in human NP cells. All results provide the pharmacological effects and mechanisms of action of baicalein, which should be considered as a potential new candidate for clinical therapy to attenuate disc degeneration. A further in vivo study will be required to verify the above in vitro experimental results.

## Supplementary Information

**Additional file 1**. Identification of nucleus pulposus cells. Nucleus pulposus cells were positive for toluidine blue staining (A), Safranin O (B) and immunohistochemistry staining of type II collagen (C)

**Additional file 2**. Baicalein modulates matrix degradation protein expression. (A) mRNA expression of MMP-2 and MMP-9 genes in NP cells treated in the absence and presence of baicalein for 6 h before TNF-α treatment (50 ng/mL), then exposed to TNF-α for 12 h. (B) Protein expression of MMP-2 and MMP-9 genes in NP cells treated in the absence and presence of baicalein for 6 h before TNF-α treatment (50 ng/mL), then exposed to TNF-α for 12 h. *P < 0.05 compared with control group, #*P* < 0.05 compared with TNF-α group.

## Data Availability

According to the requirements, data can be obtained from the corresponding author to support the results of this study.
