# Supplementary figures and images for: Baicalein alleviates TNF-α-induced apoptosis of human nucleus pulposus cells through PI3K/AKT signaling pathway
Source: J Orthop Surg Res. 2023 Apr 11;18:292. doi: 10.1186/s13018-023-03759-9 (PMC10088118; doi:10.1186/s13018-023-03759-9)

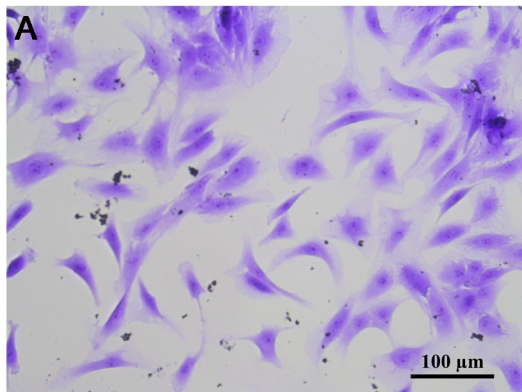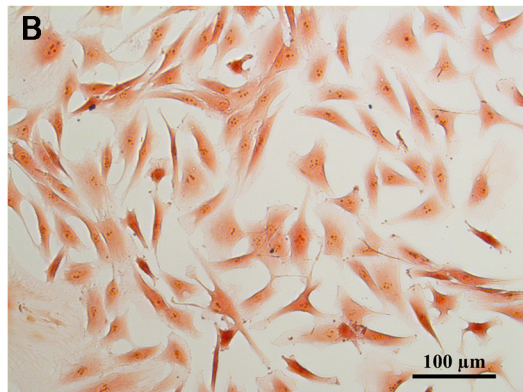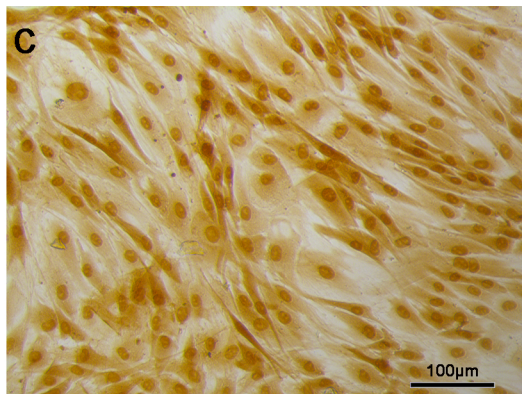

Supplement: Supplementary file 1 — Additional file 1. Identification of nucleus pulposus cells. Nucleus pulposus cells were positive for toluidine blue staining (A), Safranin O (B) and immunohistochemistry staining of type II collagen (C) [file 13018_2023_3759_MOESM1_ESM.pdf]

A

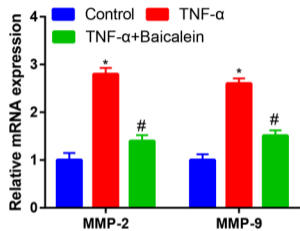

B

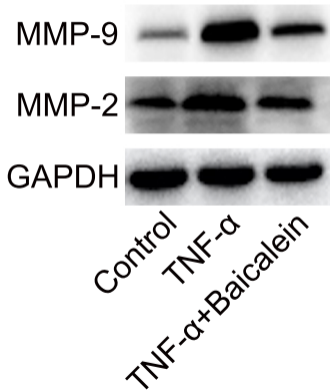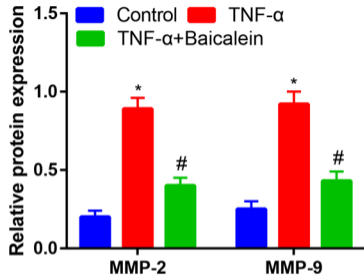

Supplement: Supplementary file 2 — Additional file 2. Baicalein modulates matrix degradation protein expression. (A) mRNA expression of MMP-2 and MMP-9 genes in NP cells treated in the absence and presence of baicalein for 6 h before TNF-α treatment (50 ng/mL), then exposed to TNF-α for 12 h. (B) Protein expression of MMP-2 and MMP-9 genes in NP cells treated in the absence and presence of baicalein for 6 h before TNF-α treatment (50 ng/mL), then exposed to TNF-α for 12 h. *P < 0.05 compared with control group, #P < 0.05 compared with TNF-α group. [file 13018_2023_3759_MOESM2_ESM.pdf]
